# Surgical treatment of gastric venous congestion in association with extended resection of pancreas: a case report

**DOI:** 10.1186/s12893-020-0692-4

**Published:** 2020-02-10

**Authors:** Shuji Kagota, Tetsunosuke Shimizu, Kohei Taniguchi, Atsushi Tomioka, Yoshihiro Inoue, Koji Komeda, Mitsuhiro Asakuma, Sang-Woong Lee, Fumitoshi Hirokawa, Kazuhisa Uchiyama

**Affiliations:** 1 Department of General and Gastroenterological Surgery, Osaka Medical College, 2-7 Daigaku-machi, Takatsuki, Osaka, 569-8686 Japan; 2 Department of Gastrointestinal Surgery, Takatsuki Red Cross Hospital, 1-1 Abuno, Yakatsiki, Osaka, 569-1096 Japan; 3 Translational Research Program, Osaka Medical College, 2-7 Daigaku-machi, Takatsuki, Osaka, 569-8686 Japan

**Keywords:** Gastric venous congestion, Gastric drainage vein, Total pancreatectomy

## Abstract

**Background:**

Total pancreatectomy is performed for chronic pancreatitis, tumors involving the entire pancreas or remnant pancreas after pancreatectomy. Gastric venous congestion and bleeding may be associated with total pancreatectomy. We report the case of a patient who underwent left gastric vein to splenic vein bypass to relieve gastric venous congestion during total pancreatectomy for remnant pancreatic cancer.

**Case presentation:**

A 60-year-old woman underwent subtotal stomach-preserving pancreaticoduodenectomy for cancer of the pancreatic head. A follow-up computed tomography revealed a low-density tumor of the remnant pancreas. The pathological diagnosis was adenocarcinoma on endoscopic ultrasound-fine needle aspiration. Total resection of the remnant pancreas was performed for the tumor 3 years after the initial surgery. We ligated the splenic vein at the point of distal side of the left gastric vein confluent. Immediately, the vein congestion around the stomach was confirmed. We found the stenosis of the confluent between the left gastric vein and splenic vein. We subsequently anastomosed the left gastric vein and splenic vein, following which the gastric venous congestion was relieved.

**Conclusion:**

In cases wherein all the drainage veins from the stomach are removed, an anastomosis between the left gastric vein and splenic vein can be effectively used to prevent gastric venous congestion and bleeding after total pancreatectomy.

## Background

Total pancreatectomy (TP) is often performed for chronic pancreatitis, the tumor of the whole pancreas, or the pancreatic remnant after pancreatectomy. TP is classified according to the need for resection or preservation of the stomach. Some reports have described gastric venous congestion and bleeding after TP, except in TP with distal gastrectomy (TPDG) [1, 2] . To avoid this, venous reconstruction and distal gastrectomy are useful. However, gastrectomy worsens the patient’s nutritional status postoperatively and affects the dynamics of the gastrointestinal hormones. When preserving the subtotal or whole stomach, preservation or reconstruction of at least one gastric draining vein is necessary.

Herein, we report a case in which a bypass from the left gastric vein (LGV) to the splenic vein (SpV) was performed to relieve gastric venous congestion during TP for cancer of the remnant pancreas.

## Case presentation

**Fig. 1:**
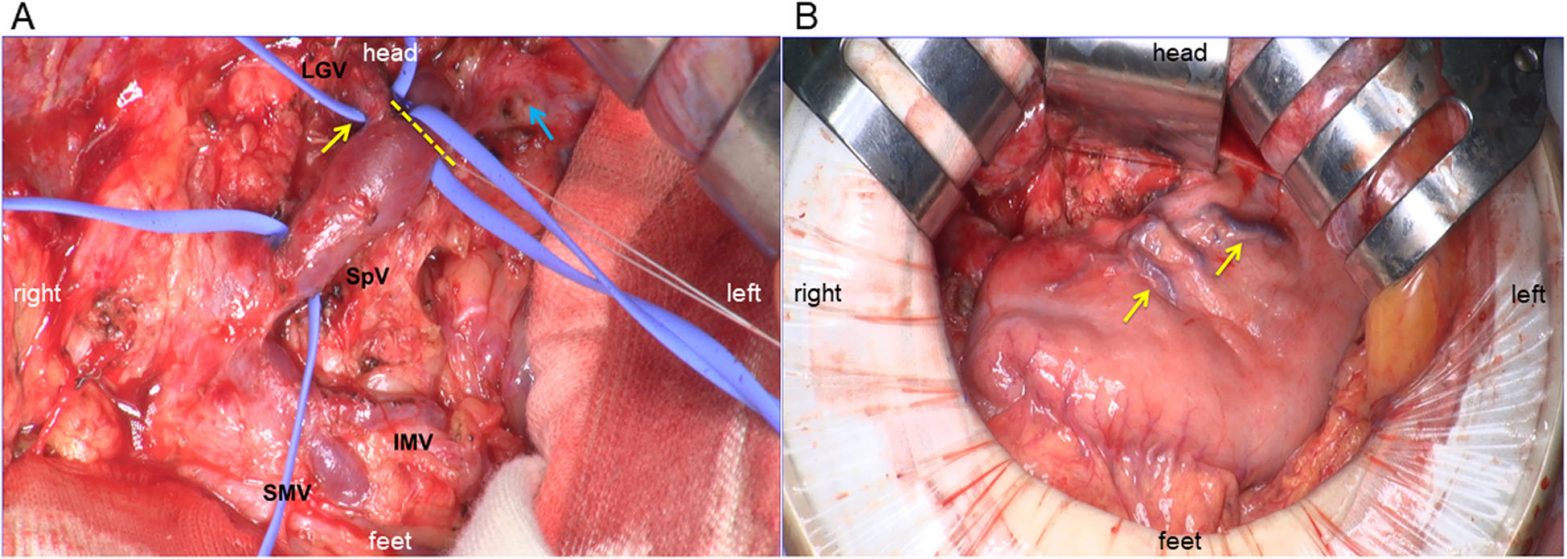
**a**. Isolation of each vein. Stenosis at the entry of the left gastric vein (LGV) to the splenic vein (SpV) (yellow arrow). The tumor near the entry of the LGV into the SpV (blue arrow). Yellow line indicates the cut line of the SpV. LGV, left gastric vein; IMV, inferior mesenteric vein; SMV, superior mesenteric vein; SpV, splenic vein. **b**. Venous congestion of the stomach at the lesser curvature (yellow arrow)

A 60-year-old female patient underwent subtotal stomach-preserving pancreaticoduodenectomy (PD) with lymphadenectomy for pancreatic head ductal adenocarcinoma. She received postoperative adjuvant chemotherapy (S-1) for 8 cycles. On 3-year follow-up after the initial surgery, abdominal contrast-enhanced computed tomography (CT) showed a 12 mm hypovascular tumor in the remnant pancreas. Endoscopic ultrasound-fine needle aspiration revealed adenocarcinoma. Although surgical indications for pancreatic cancer recurrence are controversial, we considered the possibility of de novo pancreatic cancer or pancreatic cancer recurrence. We performed total resection of the remnant pancreas. We exposed the portal vein (PV), SpV, inferior mesenteric vein (IMV), and LGV (Fig. 1a).

The tumor invaded the SpV just distal to the entry of the LGV into the SpV and dissected the SpV at this point. The splenic artery was dissected at its root by lymph node dissection. Finally, the tumor was removed. However, venous congestion at the lesser curvature of the stomach was evident (Fig. 1b); iatrogenic stenosis, which is the entry of the LGV into the SpV was revealed. The LGV and SpV were anastomosed by the end-to-end method using 6–0 nylon to allow gastric inflow into the PV. Venous congestion of the stomach at the lesser curvature was resolved.

No venous congestion was observed on CT on postoperative day 7. The patient was discharged on postoperative day 11; no recurrence was noted for 22 months after TP.

## Discussion and conclusions

Extended resection of the pancreas with portal system resection can lead to R0 resection [3]. However, TP is complicated by permanent pancreatic endocrine and exocrine deficiencies, such as postoperative diabetes mellitus and digestion disorders. Medications for controlling pancreatic secretory function have improved TP safety.

In our case, the right gastric vein and right gastroepiploic vein (RGEV) were removed during the initial surgery. The left gastroepiploic vein and short and posterior gastric veins were removed during the second operation. However, blood flow from the LGV to the SpV was insufficient owing to stenosis during the operation. The IMV and middle colic vein were preserved at the initial and second surgery. However, the arc of Barkow, which supplies the transverse colon via multiple ascending branches, was lost during splenectomy. This phenomenon is similar to sinistral portal hypertension, which causes gastrointestinal hemorrhage. Gastric congestion in our case can be attributed to the stenosis by the iatrogenic maneuver. Distal splenorenal and mesocaval shunting have been performed during complex pancreatectomy for the patients with border-line resectable or locally advanced pancreatic cancer. These techniques provide operative resection to patients with complex vascular involvement and prevent long-term potential consequences of gastric venous congestion [4].

Nakao et al. recommended TPDG to prevent venous congestion [1]. However, extended resection of the stomach with TP involves functional and structural dysfunction, resulting in worsening of the patient’s nutritional status. Tanaka et al. reported the risk for varices is dependent on the number of the gastric drainage veins preserved [5]. At least one gastric draining vein must be preserved to avoid venous congestion of the subtotal or whole stomach when performing TP. In many cases, TP loses all drainage veins to the stomach. The addition of distal pancreatectomy leads to avoid venous congestion around the lower body of the stomach. In the upper body of the stomach, venous congestion and bleeding are often avoidable because well-developed submucosal venous plexus in esophago-gastric junctions are sometimes observed.

Careful preoperative assessment for the drainage veins and meticulous operative planning and techniques to preserve them are important to minimize the risk of venous congestion in patients with locally advanced pancreatic cancer. LGV or RGEV preservation is deemed particularly important [2]. However, it may be difficult to use the RGEV in case of remnant TP because it is always removed during PD. Therefore, preserving the LGV is more important.

We are sometimes obliged to remove all draining veins from the stomach depending on the tumor size, tumor location, and invasion depth. RGEV and left ovarian vein anastomosis and LGV and IMV anastomosis were performed to effectively reduce gastric congestion. Unfortunately, the RGEV was already removed during the initial surgery in our case. The ideal anastomosis in our case was that between the LGV and stump of the SpV. The advantage of this was the short distance of each vein without tension of the anastomosis.

Our experience suggests that LGV and SpV anastomosis prevents gastric venous congestion and that gastric outflow preservation is important. It is vital that suitable revascularization be performed to avoid sinistral portal hypertension. To conclude, LGV and SpV anastomosis can be an effective option to prevent gastric venous congestion when performing TP.

## Data Availability

All data supporting the conclusions of this study are included in this published article.

## Abbreviations

IMV: Inferior mesenteric vein.
LGV: Left gastric vein
PD: Pancreaticoduodenectomy
PV: Portal vein
RGEV: Right gastroepiploic vein
RGV: Right gastric vein
SMV: Superior mesenteric vein
SpV: Splenic vein
TP: Total pancreatectomy
TPDG: Total pancreatectomy with distal gastrectomy,
